# Superplastic Deformation Behavior of Rolled Mg-8Al-2Sn and Mg-8Al-1Sn-1Zn Alloys at High Temperatures

**DOI:** 10.3390/ma13051074

**Published:** 2020-02-28

**Authors:** Shao-You Zhang, Cheng Wang, Long-Qing Zhao, Pin-Kui Ma, Jia-Wang Song, Jin Xu, Xiu-Ming Cheng, Hui-Yuan Wang

**Affiliations:** 1 State Key Laboratory of Automotive Simulation and Control, Nanling Campus, Jilin University, No. 5988 Renmin Street, Changchun 130025, China; 2 Key Laboratory of Automobile Materials of Ministry of Education & School of Materials Science and Engineering, Nanling Campus, Jilin University, No. 5988 Renmin Street, Changchun 130025, China; 3 International Center of Future Science, Jilin University, Changchun 130012, China

**Keywords:** superplasticity, grain boundary sliding, microstructural evolution, Mg_2_Sn particles

## Abstract

The high-temperature superplastic deformation behavior of rolled Mg-8Al-2Sn (AT82) and Mg-8Al-1Sn-1Zn (ATZ811) alloys were investigated in this study. During tensile deformation at 573 K, no obvious grain growth occurred in both alloys, because of the high-volume fraction of second phases located at grain boundaries. Meanwhile, texture weakening was observed, suggesting that grain boundary sliding (GBS) is the dominant superplastic deformation mechanism, which agreed well with the strain rate sensitivity (*m*) and the activation energy (*Q*) calculations. The microstructural evolution during tensile deformation manifested that there were more and larger cavities in AT82 than ATZ811 during high-temperature tensile deformation. Therefore, superior superplasticity was found in the ATZ811 alloy that presented a tensile elongation of ~510% under a strain rate of 10^−3^ s^−1^ at 573 K, in contrast to the relatively inferior elongation of ~380% for the AT82 alloy. Meanwhile, good tensile properties at ambient temperature were also obtained in ATZ811 alloy, showing the ultimate tensile strength (UTS) of ~355 MPa, yield strength (YS) of ~250 MPa and elongation of ~18%. Excellent mechanical performance at both ambient and elevated temperatures can be realized by using economical elements and conventional rolling process, which is desirable for the industrial application of Mg alloy sheets.

## 1. Introduction

As the lightest metallic structural materials among those developed thus far, magnesium (Mg) and its alloys have been developing as one of the promising materials in the aerospace and automotive industries [1,2,3]. However, the poor formability at room temperature leads to significant difficulties in processing and application of Mg alloys, which results from the intrinsic nature of hexagonal close-packed (hcp) crystals [4]. High-temperature superplastic deformation (elongation to failure of ≥ 500% [5]) is considered as a good choice for forming Mg-based products for automobile components with complex shapes. Therefore, it is of great importance to attain novel Mg alloys with high superplasticity, and study their deformation behaviors at elevated temperatures.

The Mg-Al based alloys are currently the most widely used Mg alloys because of their low cost and relevantly good properties [6], in particular for the Mg-Al-Zn alloys consisting of α-Mg matrix and β-Mg_17_Al_12_ strengthening phases [7]. The softening of low melting-point Mg_17_Al_12_ phase at elevated temperatures facilitates the interphase boundary sliding, and thus assists in developing their superplasticity [8]. Several reported studies have achieved the high-temperature superplasticity in Mg-Al-Zn alloys by friction stir processing (FSP) [9], high ratio differential speed rolling (HRDSR) [10], and controlled rolling [11,12]. However, the room-temperature strength of commercially available Mg-Al-Zn wrought Mg alloys are still difficult to meet the industrial demand [13,14]. Therefore, there is an urgent need to develop new wrought Mg alloys with remarkable mechanical properties at ambient and high temperatures and expand their commercial applications.

Recent studies show that Sn element is not just a cheap alternative of rare earth (RE) elements, and it can also effectively improve the strength of Mg alloys [15,16,17]. The Mg-Al-Sn have been proved to have excellent mechanical properties at ambient temperature [15]. However, excessive Sn addition can result in the formation of coarse undissolved Mg_2_Sn particles [18], which significantly reduces the ductility of the final products. The coarse undissolved Mg_2_Sn particles are difficult to relieve by conventional processes that are suitable for industrial-scale production. Therefore, a suitable Sn addition should be taken into account to suppress the formation of coarse undissolved Mg_2_Sn particles.

Moreover, according to our previous work [18], Zn element is considered to be an effective addition agent for further improving the mechanical properties of Mg-Al-Sn alloys, and the Mg-Al-Sn-Zn based alloys with a high Al (~8 wt %) and a low Sn content have been proved to have excellent mechanical properties at ambient temperature [18]. Meanwhile, this alloy system could have a good superplastic potential because of their high volume fraction of low-melting second phase (Mg_17_Al_12_) and fine particles (Mg_2_Sn) with good thermal stability. However, to date, there have been few reports on the high-temperature superplasticity of the Mg-Al-Sn-Zn-based alloys.

In the present study, the Mg-8Al-2Sn (AT82) and Mg-8Al-1Sn-1Zn (ATZ811) alloys were chosen as the experimental materials, and the fine-grained structures were obtained in both alloys via a controlled 13-pass rolling. The effect of the substitution of 1 Zn for 1 Sn (wt %) in AT82 alloy was analyzed by investigating microstructure evolution and superplastic deformation behavior. The study aims to develop a new wrought Mg alloy with excellent performance at both ambient and elevated temperatures by using economical elements and conventional rolling process, which is desirable for the industrial application of Mg alloy sheets. The results of this work may provide a valuable reference for composition design and controlled fabrication of lightweight Mg-based sheet products for automobile covering parts.

## 2. Materials and Methods

**Figure 1:**
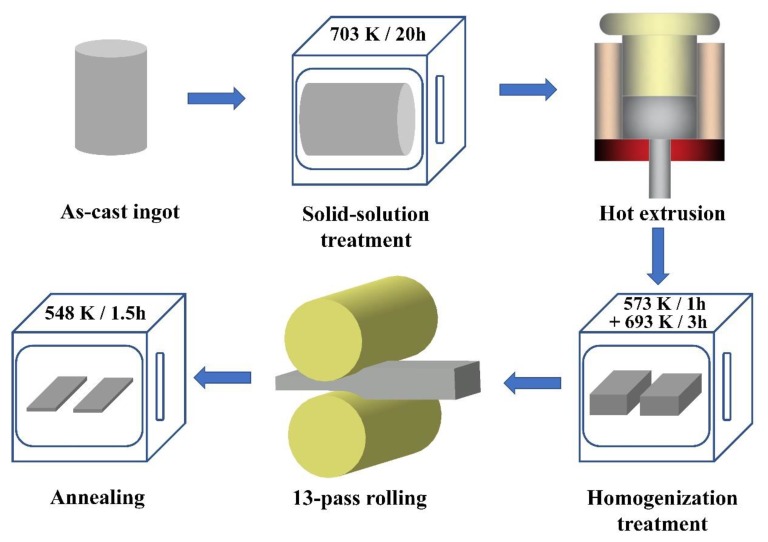
Schematic illustration showing the methods used in this work.

The as-cast Mg-8Al-2Sn (wt %, AT82) and Mg-8Al-1Sn-1Zn (wt %, ATZ811) alloys were prepared from commercial pure Mg (99.85 wt %, purity), pure Al (99.90 wt %), pure Sn (99.90 wt %), and pure Zn (99.90 wt %) by an electrical resistance melting furnace under a mixture of protective SF_6_ and CO_2_ at ~953 K. After being purified and kept for about 15 min, the melts were poured into a steel mold (pre-heated to about 323 K) with a cylindrical cavity of 90 mm in diameter and 200 mm in height. The actual chemical compositions of the studied alloys measured by an optical spectrum analyzer (ARL 4460, Ecublens, Switzerland) are Mg-8Al-1.9Sn (wt %) for AT82 and Mg-7.9Al-1.1Sn-1Zn (wt %) for ATZ811. A solution treatment was conducted on the ingots at 703 K for 20 h to dissolve parts of the second phase into matrix. Subsequently, the ingots were hot extruded at ~663 K under a high-ratio of 33:1 to obtain slabs with a thickness of 5 mm. Rolling samples with dimensions of 5 mm (thickness) × 30 mm (length) × 20 mm (width) were cut from the extruded slabs and homogenized at 573 K for 1 h and then 693 K for 3 h to remove residual stress prior to rolling experiments. The samples were subjected to a hot rolling with 13 rolling passes and a total reduction of ~76.5% under descending temperature, ranging from 623 to 573 K to obtain the final sheets with a thickness of 1.1 mm. Note that the rolling reduction per pass was ~10% and the rollers were heated to 373 K during the whole process. Eventually, the rolled sheets were annealed at 548 K for 1.5 h to optimize the microstructure. For a better understanding, the schematic illustration showing the methods used in this work is given in Figure 1.

To estimate the tensile properties of the rolled AT82 and ATZ811 alloys, tensile samples with a gauge size of 10 mm (length) × 4 mm (width) were machined from the rolled sheets with the loading axis parallel to the rolling direction (RD). The tensile tests at room temperature were carried out on an AGS-X-100kN electric universal testing machine (SHIMADZU, Suzhou, China) with a strain rate of 1.0 × 10^−3^ s^−1^. At least four samples were tested, and the stress–strain curves with good repeatability were produced. The high-temperature tensile tests were performed under constant-crosshead-speed conditions on an AGS-X-10kN electric universal testing machine (SHIMADZU, Suzhou, China) with a strain rate of 1.0 × 10^−3^ s^−1^ at various temperatures between 423 K and 573 K. Meanwhile, the strain rate change (SRC) tests were carried out in the crosshead speed ranging from 0.6 to 0.9 mm min^−1^ (1.0 × 10^−3^ s^−1^ and 1.5 × 10^−3^ s^−1^ in initial strain rate) at 573 K to measure the value of strain-rate sensitivity (m). A pre-strain of ε = 0.15 was imposed at an initial strain rate of 1.0 × 10^−3^ s^−1^ before the SRC was applied to stabilize the microstructure, and then a strain of ~0.05 was applied between the strain rates of 1.0 × 10^−3^ s^−1^ and 1.5 × 10^−3^ s^−1^.

The phase constituents of the investigated alloys were examined by X-ray diffraction (XRD; Model D/Max 2500PC Rigaku, Tokyo, Japan) using Cu Kα radiation at a voltage of 40 kV with a scanning speed of 2° min^−1^. Microstructural characterization was performed with a scanning electron microscope (SEM; ZEISS EVO18, Göttingen, Germany) equipped with an energy dispersive spectrometry (EDS; INCA-X-Max, Cambridge, UK). The metallographic samples were taken from the planes perpendicular to the normal direction (ND). The samples for SEM observations were first ground with 2000 mesh SiC paper, buffed with 0.5 μm diamond paste, and then chemically etched in an acetic picric solution (5 g picric acid, 5 mL acetic acid, 10 mL distilled water, and 80 mL ethanol) for ~20 s. Electron backscatter diffraction (EBSD) analysis was carried out in a scanning electron microscope (VEGA 3 XMU, TESCAN, Brno, Czechoslovakia) equipped with an Oxford Instruments Nordlys Nano EBSD detector that used AZtec and Channel 5.0 software (Aztec Software Private Limited, Bengaluru, Karnataka) to collect and analyze the data. The EBSD analysis was performed at 20 kV with a 20 mm working distance, a 70° tilt and 0.5 µm scan steps. The average size and distribution of grains and precipitates were obtained by the Nano Measurer 1.2, and the area fraction of the second phase was estimated by the ImageJ, which were derived from at least eight micrographs.

## 3. Results and Discussion

### 3.1. Microstructural Characteristics of As-Rolled Materials

**Figure 2:**
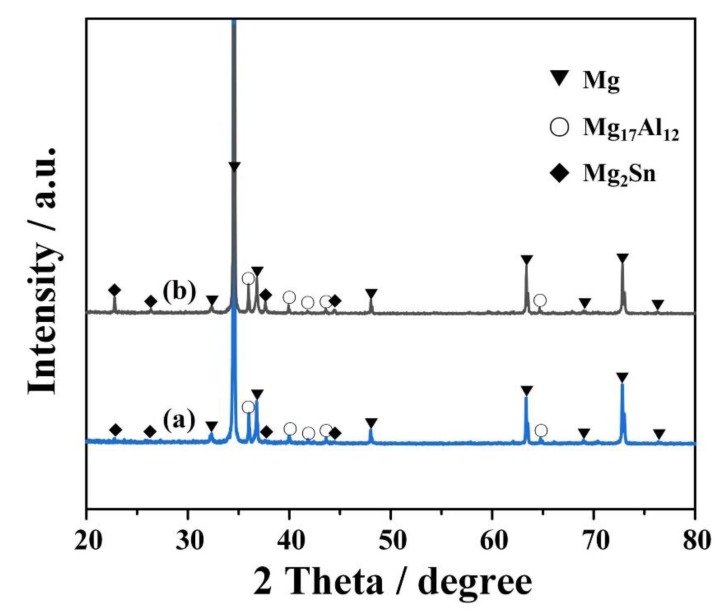
X-ray diffraction pattern showing the phase composition of (**a**) AT82 and (**b**) ATZ811 alloys after 13-pass rolling (~76% thickness reduction).

The XRD results in Figure 2 reveal that the α-Mg, Mg_17_Al_12_ and Mg_2_Sn phases are detected in both the AT82 and ATZ811 alloys. It is worth noting that no Mg-Al-Zn ternary phase is found in the ATZ811 alloy, because Mg-Al-Zn phases only form when the Zn/Al ratio is >3 [19]. Additionally, no Zn-containing phases are detected, presumably because of its high solubility in Mg [20] and the relatively minor quantity in this work.

**Figure 3:**
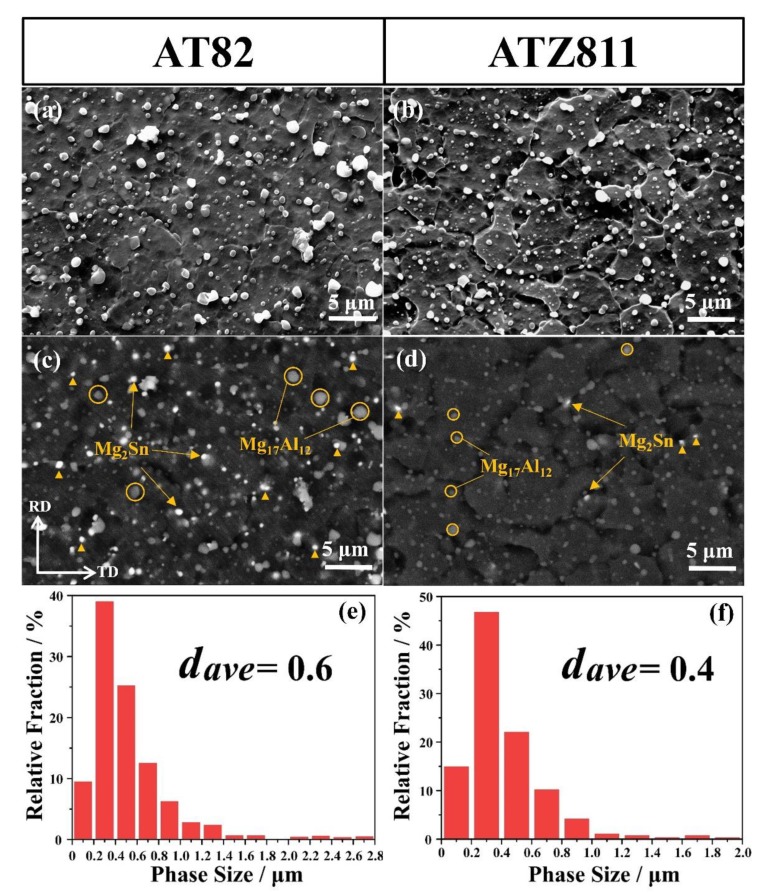
(**a**,**b**) Scanning electron microscope (SEM), (**c**,**d**) corresponding backscatter scanning electron microscopy (BSEM) images and (**e**,**f**) size distribution of the second phases, showing a comparison of the precipitate features in the rolled and annealing alloys. Here, (**a**,**c**,**e**) correspond to AT82 alloy, and (**b**,**d**,**f**) correspond to ATZ811 alloy. The *d_ave_* represents the average size of the second phases in each sample.

Figure 3a,b shows the microstructures of the rolled and annealed AT82 and ATZ811 samples. Both of the two cases exhibit a fully recrystallized characteristic with fine equiaxed grains, showing fine and nearly spherical precipitates that were uniformly dispersed in the matrix. This is a typical morphology obtained by multi-pass controlled rolling as reported by previous works [12,21], which is due to the thermodynamic coupling and the Ostwald ripening effect during hot rolling and inter-pass annealing. The corresponding backscatter scanning electron microscopy (BSEM) images are given in Figure 3c,d. It is clear that the phases in both AT82 and ATZ811 alloys have two distinct features, which is consistent with the XRD results in Figure 2. According to the EDS results, the light-grey particles with relatively big size are Mg_17_Al_12_ (marked by circular symbols), while the bright particles with relatively fine size are Mg_2_Sn (marked by triangle symbols), which agrees with the theory of BSEM analysis that atoms with higher Z values have a brighter color.

Upon comparing the microstructures of AT82 (Figure 3a,c) and ATZ811 (Figure 3b,d), as expected, there are significantly more Mg_2_Sn particles in the former material than those in the latter. Meanwhile, the amount of Mg_17_Al_12_ phase in ATZ811 is much greater than that in AT82, because the addition of Zn reduces the solid solubility of Al and thus promotes the precipitation of Mg_17_Al_12_ phases [22,23]. Additionally, the precipitates in ATZ811 exhibit a more uniform distribution, and the size of Mg_17_Al_12_ is obviously finer than those in AT82. As shown in Figure 3e,f, the precipitate sizes in ATZ811 present a relatively narrower distribution range, mostly valuing under ~2 μm, while there are some coarse precipitates sized in ~3 μm in AT82. This feature of second phases agrees well with the previous study, which stated that the Mg_17_Al_12_ particles would increase in amount and simultaneously decrease in size when adding Zn element to the Mg alloys [18,24].

**Figure 4:**
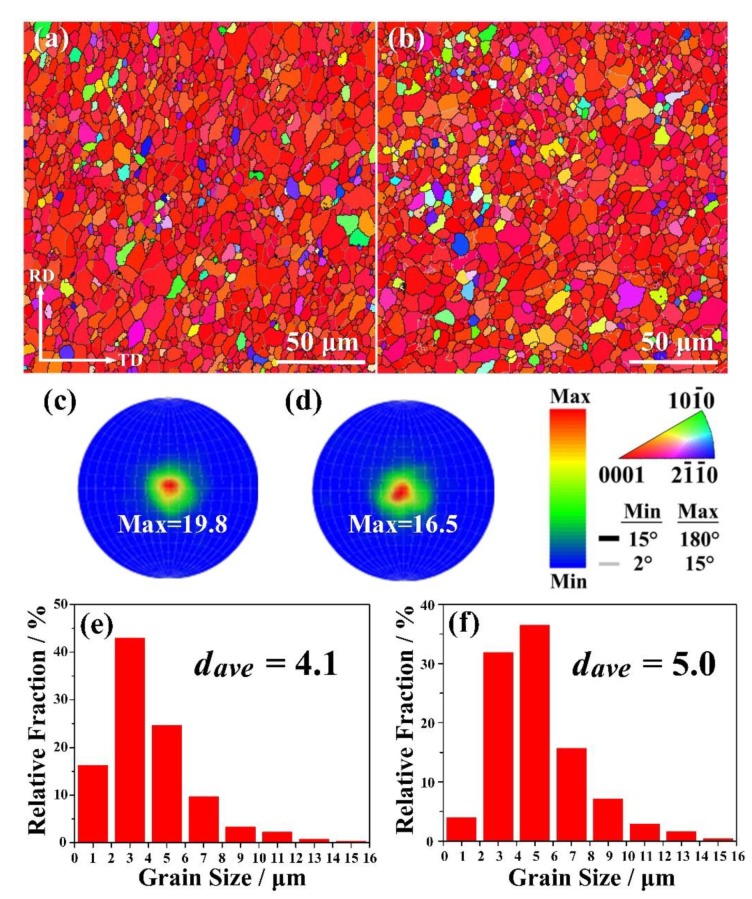
(**a**,**b**) Electron backscatter diffraction (EBSD) orientation maps, (**c**,**d**) corresponding microscopic (0002) pole figures, and (**e**,**f**) grain size distribution of the rolled and annealed alloys. Here, (**a**,**c**,**e**) correspond to AT82 alloy, and (**b**,**d**,**f**) correspond to ATZ811 alloy. The number in each pole figure indicates the maximum texture intensity, and the *d_ave_* represents the average grain size of each sample.

The EBSD orientation maps, along with the corresponding microscopic (0002) pole figures and grain size distributions of the rolled and annealed samples are shown in Figure 4. The average grain size of AT82 is slightly finer than that of ATZ811. According to the grain size distribution in Figure 4e,f, it reveals that the majority of grains in AT82 exhibit a narrower range of 1–7 μm than that in ATZ811 (3–9 μm). The difference in grain size is because that there are more Mg_2_Sn particles with high thermal stability in AT82 compared with ATZ811. During the hot rolling and inter-pass annealing process, the Mg_17_Al_12_ phases precipitate and dissolve repeatedly accompanied by softening and coarsening due to their low thermal stability [11,25], leading to a relatively poor effect on hindering grain growth. However, the presence of fine and homogeneously dispersed Mg_2_Sn precipitates along the grain boundaries can be quite beneficial to obtaining a fine-grain microstructure. As shown in Figure 4c,d, both of the two samples exhibit a strong basal texture, revealing that the c-axes of most grains align parallel to the sheet normal direction, which is a typical texture in rolled Mg alloys with high Al content [26,27]. Note that the micro-texture of ATZ811 is slightly weaker than that of AT82, which is probably because the addition of Zn facilitates the recrystallization by the particle stimulated nucleation (PSN) mechanism, as reported by Wang et al. [18].

### 3.2. Mechanical Properties at Ambient Temperature (298 K)

**Figure 5:**
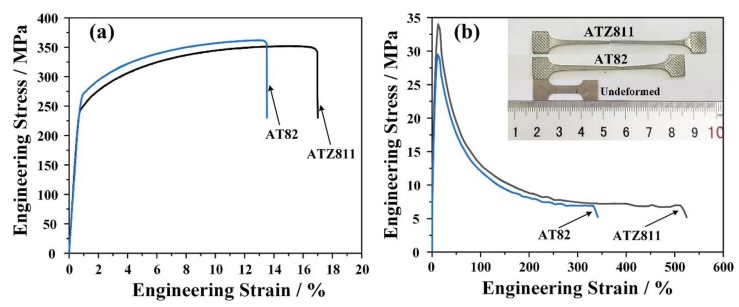
Tensile engineering stress–strain curves of the rolled and annealed sheets at (**a**) room temperature (298 K) and (**b**) 573 K with a strain rate of 10^−3^ s^−1^. The upper right inset compares the macrographs before and after high-temperature tensile deformation.

The typical tensile engineering stress–strain curves of the rolled and annealed sheets are shown in Figure 5, with the corresponding tensile properties listed in Table 1. The substitution of 1 wt % Zn for 1 wt % Sn results in an increase in elongation of the AT82 alloy from ~14 to ~18%, accompanied by a decrease in the ultimate tensile strength (UTS) and yield strength (YS), from ~366 MPa to ~355 MPa and ~270 MPa to ~250 MPa, respectively. The better tensile elongation of ATZ811 is attributed to the more uniformly dispersed and finer precipitates compared with AT82 (Figure 3). It is well known that coarse second phase particles can degrade the ductility of alloys. The coarse precipitates could cause stress concentration and become the source of cracks during tensile deformation, consequently inducing early fracture.

It is well-known that fine-grain strengthening is one of the dominant factors responsible for the enhanced YS of Mg alloys. As mentioned above, the average grain size in AT82 is slightly finer than that in ATZ811, and the majority of grains in AT82 also exhibit a narrower range (Figure 4e,f). The increment in YS by grain-boundary strengthening (△*σ*_grain_) can be estimated using a standard Hall-Petch equation:

△σ_grain_ = *kd*^−1/2^ (1)

where *d* is the average grain size and *k* is the Hall-Petch coefficient (0.29 MPa m^0.5^ for pure Mg [28]). Therefore, taking *d* of ~4.1 μm and ~5.0 μm into Equation (1), the grain-boundary strengthening can account for an increment of approximately 143 MPa and 130 MPa for the AT82 and ATZ811 alloys, respectively. The calculated values indicated that the difference of YS between the two alloys was ~13 MPa, which is less than the measured YS difference. It is as expected because there is another part of the increment that resulted from the increasing Mg_2_Sn precipitates.

More fine particles can act as barriers to dislocation motion, which is known to enhance the YS by Orowan mechanism. The precipitation strengthening (△*σ_PPT_*) can be estimated on the basis of the Orowan looping mechanism. Nie et al. developed an Orowan equation which focuses specifically on the spherical precipitates in Mg alloys [29]. According to the proposed model, the increment in YS due to Orowan strengthening, is expressed as:

(2) $$△{\sigma_{\mathrm{PPT}}}_{\;}=\;M\frac{Gb}{2\pi \sqrt{1-v\;}\left(\frac{0.779}{\sqrt{f}}\;-\;0.785\right)d_{t}}ln\frac{0.785d_{t}}{b}$$

where *M* is the Taylor factor. The values are between 2.1 and 2.5 for polycrystalline Mg alloy with textures that inhibit basal and prismatic slip while favoring pyramidal slip. As the rolled sheets in this work show the basal texture, a value of *M* = 2.3 may be assumed [30]. *G* is the sheer modulus (1.66 × 10^4^ MPa for Mg [30]), *b* is the Burgers vector (3.2 × 10^−10^ m for Mg [30]), *v* is the Poisson’s ratio of Mg (0.35 [30]), *d_t_* is the mean radius of a circular cross section in a random plane for the spherical precipitate, and *f* is the volume fraction of precipitates. In this work, the value of *d_t_* is estimated to be 3 × 10^−7^ m in AT82 and 2 × 10^−7^ m in ATZ811; and the value of *f* is estimated to be ~0.25 in AT82 and ~0.16 in ATZ811. According to Equation (2), dispersion strengthening can account for the increment of approximately ~69 MPa and ~63 MPa for the AT82 and ATZ811 alloys, respectively. The calculated values indicated that the difference in YS resulted from precipitation strengthening between the two alloys was ~6 MPa. As mentioned above, the size of Mg_17_Al_12_ particles in ATZ811 alloy is even smaller than that in AT82 alloy, therefore the larger precipitation strengthening in AT82 alloy than ATZ811 alloy is mainly due to more Mg_2_Sn particles.

By summing the increments from the grain boundary strengthening and precipitation strengthening, the YS of AT82 is ~20 MPa larger than that in ATZ811 alloy. The calculated value is almost consistent with the measured difference value. It can be concluded that the YS in AT82 alloy is higher than that of ATZ811 alloy because of the cooperation effect of grain boundary strengthening and precipitation strengthening from Mg_2_Sn particles.

### 3.3. Mechanical Properties at the High Temperature (573 K)

The engineering stress–strain curves of the rolled and annealed AT82 and ATZ811 sheets tested at 573 K under a strain rate of 10^−3^ s^−1^ are presented in Figure 5b, and corresponding tensile properties are summarized in Table 1. Clearly, the ATZ811 alloy has a great advantage in superplasticity with the maximum fracture strain of ~510%, in contrast, the value of AT82 is only ~380% under the same condition. A good high-temperature performance in the Mg-Al-Sn-Zn alloy sheets was achieved, which benefits from the fine equiaxed grain structure and uniformly dispersed precipitates obtained by the controlled rolling process.

Moreover, upon comparing the Figure 5a,b, it can be seen clearly that the increase of deformation temperature can decrease the strength extremely. At 573 K, both alloys exhibit a significant drop in strength compared with the ambient temperature. Ferdous et al. [31] reported the similar phenomenon in the polymer concrete, and the mechanism behind this was explained as the loss of internal resistance at high temperatures. In this work, the decrease of deformation resistance at high temperatures could be attributed to the activation of additional deformation modes, such as grain-boundary sliding and non-basal slips, which is beneficial to the plastic deformation and result in the decreased strength in both alloys.

#### 3.3.1. Superplastic Deformation Mechanism at 573 K

**Figure 6:**
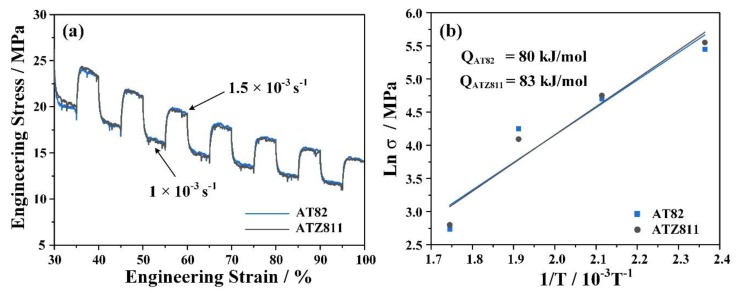
(**a**) Engineering stress–strain curves of AT82 and ATZ811 alloys obtained from the SRC tests at strain rates between 1 × 10^−3^ and 1.5 × 10^−3^ s^−1^ to calculate the strain rate sensitivity (*m* value); (**b**) the variation of *lnσ* as a function of 1/*T* at the strain rate of 1 × 10^−3^ s^−1^ to calculate the deformation activation energy (*Q* value).

The engineering stress–strain curves obtained from the SRC tests for rolled and annealed AT82 and ATZ811alloys under strain rates of 1 × 10^−3^ s^−1^–1.5 × 10^−3^ s^−1^ are given in Figure 6a. The results show that there are no disparities in SRC curves between the two alloys, and the average values of the strain rate sensitivity (*m*) measured from the SRC tests are quite close, valuing ~0.52 and ~0.50 for AT82 and ATZ811, respectively. Generally, the m value of 0.50 is considered as a critical value that grain boundary sliding (GBS) will become the predominant deformation mechanism, leading to excellent superplasticity at high temperature [1,32,33]. Additionally, the values of deformation activation energy (*Q*) were calculated from the equation:

(3) $$Q=\frac{R}{m}{\frac{\partial \ln\sigma }{\partial \left(1/T\right)}|}_{\dot{\varepsilon }}$$

where *R* is the gas constant, *σ* is the flow stress, and *T* is the temperature. The relationship between *lnσ* and 1/*T* is shown in Figure 6b. The calculated *Q* values of AT82 and ATZ811 alloys are 80 and 83 kJ/mol, respectively, both of which are close to that of GB self-diffusion in pure Mg (92 KJ/mol) [34]. Therefore, the dominant superplastic deformation mechanism of the two alloys in the present work is the GBS controlled by GB self-diffusion. This result is consistent with the deformation mechanism maps for Mg alloys at elevated temperatures (573–673K) proposed by Kim et al. [35].

As analyzed above, the deformation mechanism is not the cause of the superplastic difference between the two alloys. Therefore, to further explore this phenomenon, the microstructural evolution during the tensile deformation at 573 K was observed and analyzed in detail.

#### 3.3.2. Microstructural Evolution during the Tensile Deformation at 573 K

**Figure 7:**
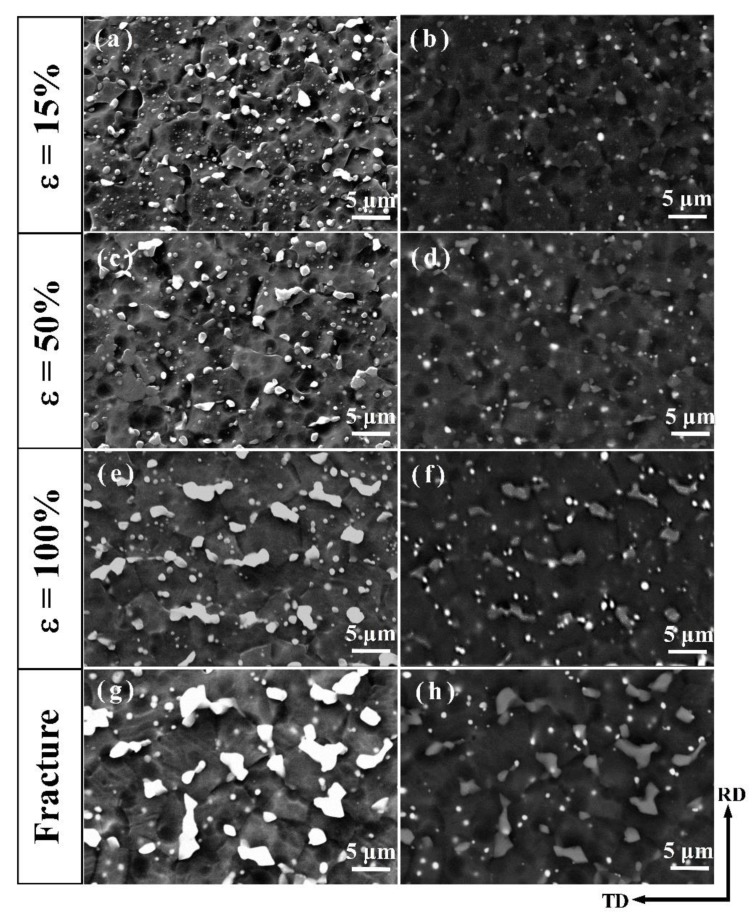
The interrupted SEM images and corresponding BSEM images of the AT82 alloy showing the evolution of the second phases during the tensile test at 573 K. Hereinto, (**a**,**b**), (**c**,**d**), (**e**,**f**), and (**g**,**h**) correspond to the elongation of ∼15%, ∼50%, ∼100%, and the fracture (i.e., ∼380% for AT82), respectively.

**Figure 8:**
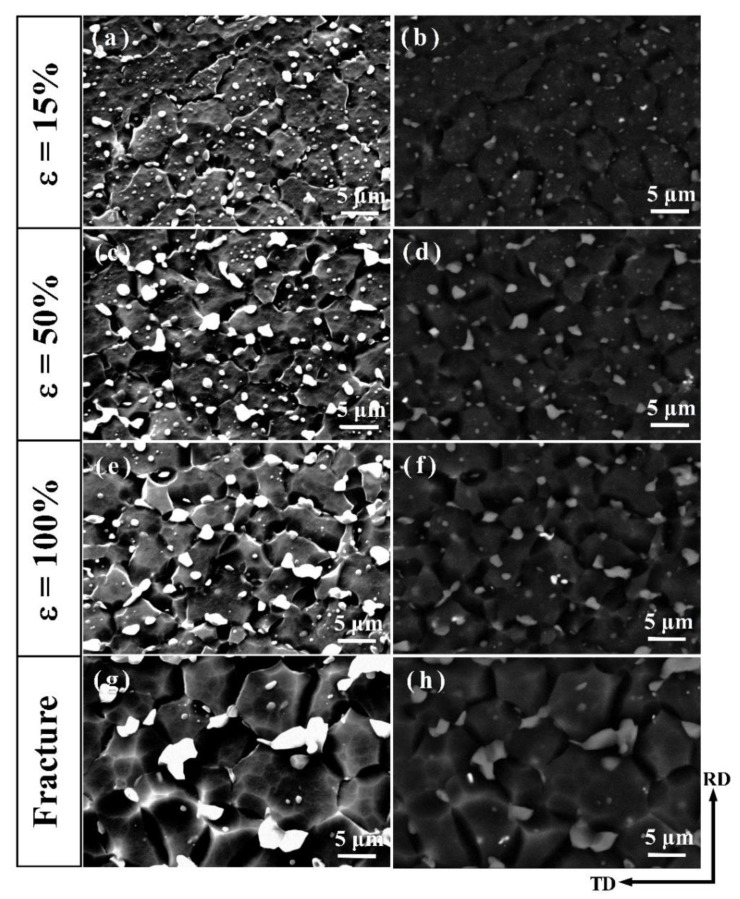
The interrupted SEM images and corresponding BSEM images of the ATZ811 alloy showing the evolution of the second phases during the tensile test at 573 K. Hereinto, (**a**,**b**), (**c**,**d**), (**e**,**f**), and (**g**,**h**) correspond to the elongation of ∼15%, ∼50%, ∼100%, and the fracture (i.e., ∼510% for ATZ811), respectively.

The truncated deformation microstructure for AT82 and ATZ811 alloys are presented in Figure 7 and Figure 8. In the interrupted tensile tests for each alloy (AT82 and ATZ811), microstructures at different deformation stages (15%, 50%, 100%, and failure elongation) were taken from the same equivalent zones of four tensile specimens, and the observation area was chosen in the gauge section close to fracture. Hereinto, the ~15% strain corresponds to the peak flow stress, and the failure elongation were ∼380% for the AT82 alloy and ∼510% for the ATZ811 alloy. In contrast to the dispersive particles in the rolled samples (Figure 3), coarsening and coalescence of Mg_17_Al_12_ particles occur gradually during the high-temperature tensile process in both of the two alloys. Note that the Mg_2_Sn particles do not show significant variation in size and quantity, which is attributed to their good thermal stability. For the AT82 alloy, the Mg_17_Al_12_ particles do not go through significant coarsening at the strain of ~15% and ~50% (Figure 7a–d), while when the strain reaches ~100%, serious coarsening of second phases occur along grain boundaries, evolving into segregated particles in irregular morphology (Figure 7e,f). This phenomena continues until fracture (Figure 7g,h), which is similar to the reported study on the AZ91 alloy [11]. While for the ATZ811 alloy, the Mg_17_Al_12_ particles slightly coarsen at the strain of ~50% without obvious coalescence (Figure 8c,d), and even until the fracture, there is only slight coalescence observed (Figure 8e–h). Upon comparing results from different work, the coalescence of Mg_17_Al_12_ occurs in the AZ91 [11] and AT82 alloys, but not in the ATZ811 alloy. This suggests that inhibition of the Mg_17_Al_12_ coalescence may be due to the cooperative effect of Sn and Zn elements. On one hand, it is well-known that the coarse and coalescent particles can cause a stress concentration and induce early fracture. Therefore, it seems to explain the better elongation of ATZ811 than that of AT82 reasonably. However, on the other hand, the softened Mg_17_Al_12_ particles segregating along the grain boundaries may accommodate intergranular incompatibilities during deformation at elevated temperatures [11], thus resulting in a good elongation. Therefore, the feature of Mg_17_Al_12_ phase is not a reasonable explanation for the superplastic differences between AT82 and ATZ811, and further analysis should be focused on another phase, i.e., Mg_2_Sn.

**Figure 9:**
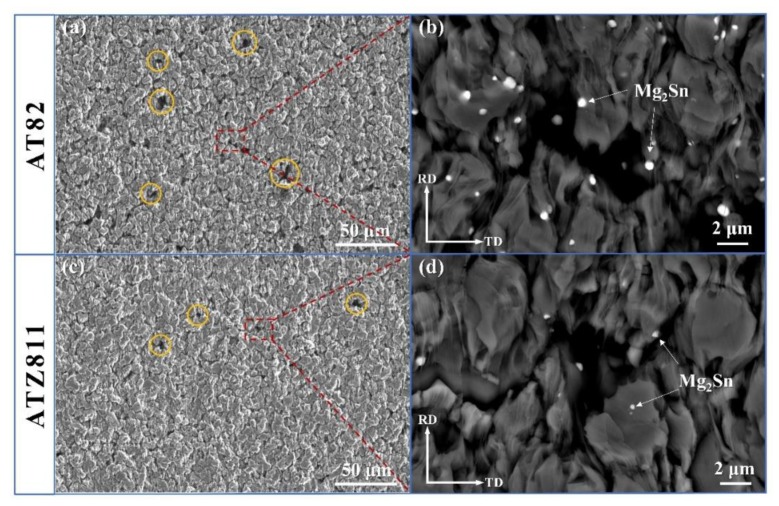
The interrupted SEM images for the (**a**) AT82 and (**b**) ATZ811 alloys subjected to tensile tests at 573 K with the elongation of ~300% showing the cavities morphology, and (**c**,**d**) correspond to the high magnification BSEM images of the red dotted boxes in (**a**,**b**), respectively, showing the Mg_2_Sn particles distribution.

The SEM images of the AT82 and ATZ811 alloys subjected to tensile tests at 573 K with the elongation of ~300% are given in Figure 9a,c. Note that the observation area is the gauge section surface without chemical etching to better demonstrate the morphology of cavities. Clearly, at the same strain of ~300%, there are obviously more and larger cavities in AT82 than those in ATZ811. There is no doubt that the cavities will expand and cause cracks, thus further result in fracture in the subsequent deformation, which can be the main reason for the inferior superplasticity of AT82 compared to ATZ811. To analyze the causes of different morphology of cavities, the corresponding high magnification BSEM images of local area (marked by red dash box) in Figure 9a,c are shown in Figure 9b,d. According to the results, there are Mg_2_Sn particles around the boundary of cavities in both cases. Therefore, it is reasonable to assume that the existence of Mg_2_Sn particles promotes the formation of cavities under high-temperature tensile tests. The small and rigid Mg_2_Sn particles would impede the GBS, and thus detrimentally induce cavity formation. Besides, the cavities would be more likely to form near the rigid particles during tensile deformation at elevated temperatures, because of the incompatible deformation between second phases and matrix. The substitution of 1 Zn for 1 Sn (wt %) in AT82 alloy results in a decreasing amount and a better uniformity of Mg_2_Sn particles. Therefore, compared to AT82 alloy, the cavities are more difficult to form in ATZ811 alloy, thus leading to an excellent superplasticity.

**Figure 10:**
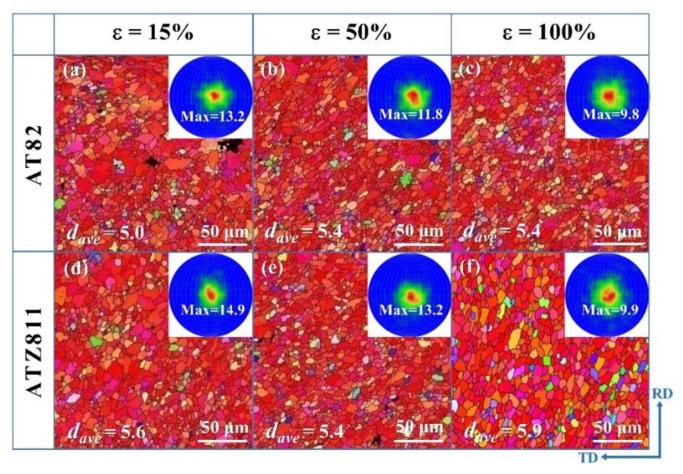
The interrupted EBSD orientation maps and corresponding microscopic (0002) pole figures of the AT82 and ATZ811 alloys showing the evolution of grain and texture during the tensile test at 573 K. Hereinto, (**a**,**d**), (**b**,**e**), and (**c**,**f**) correspond to the elongation of ∼15%, ∼50%, and ∼100%, respectively. The number in each pole figure indicates the maximum texture intensity, and the *d_ave_* represents the average grain size of each sample.

Figure 10 shows the EBSD orientation maps and corresponding microscopic (0002) pole figures of the AT82 and ATZ811 alloys in the gauge section at strains of ~15% (the peak flow stress), ~50%, and ~100%, respectively. At the interrupted strain of ~15%, the equiaxed grain structure is observed in gauge section of both alloys (Figure 10a,d), but the uniformity is not as good as the condition before tensile deformation (Figure 4a,b). As the strain increases to ~50% (Figure 10b,e) and further to ~100% (Figure 10c,f), the grains gradually become more and more uniform during the tension process, and there was no obvious grain growth in both the alloys. Additionally, according to the analysis of the corresponding microscopic (0002) pole figures, the micro-structure intensity decreases gradually during the high-temperature tensile deformation, which also verifies the activation of GBS.

**Figure 11:**
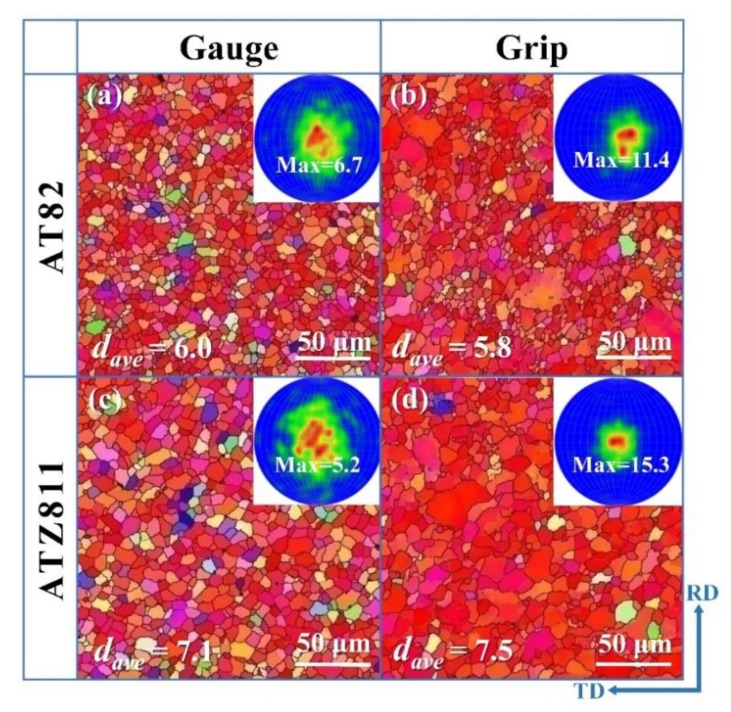
The EBSD orientation maps and corresponding microscopic (0002) pole figures of the fractured samples (i.e., ∼380% for AT82 and ∼510% for ATZ811). Hereinto, (**a**,**c**) correspond to the tensile section and (**b**,**d**) correspond to the grip section. The number in each pole figure indicates the maximum texture intensity, and the *d_ave_* represents the average grain size of each sample.

The EBSD orientation maps and corresponding microscopic (0002) pole figures of the fractured samples, i.e., ∼380% for AT82 and ∼510% for ATZ811, are given in Figure 11. Hereinto, Figure 11a,c correspond to the gauge section and Figure 11b,d corresponds to the grip section which can be considered as the part without tensile deformation. The gauge section has an obviously more uniform and finer grain structure than that of the grip section in both alloys. The formation of finer grains in the gauge section can be attributed to the dynamic recrystallization (DRX) that occurs during tensile deformation at elevated temperatures.

Both of the grip and gauge sections exhibit a relatively weaker micro-texture than that in the rolled sheets (Figure 4c,d), indicating that recrystallization occurs at this temperature. Furthermore, the gauge section shows a much weaker micro-texture compared to the grip section. The grip section still exhibits a basal texture (Figure 11b,d), while the gauge section tends to evolve into a spreading one (Figure 11a,c). There are probably two main reasons for the texture weakening during high-temperature tensile deformation. One is due to the recrystallization and the other is because of the rotation of crystallographic orientation induced by GBS [1,36]. However, because of the strong basal texture is still observed in the grip section where complete recrystallization occurs, it is reasonable to conclude that recrystallization cannot solely reduce the texture intensity dramatically. The significant randomization and weakening of texture in the gauge section can be attributed to the rotation of crystallographic orientation induced by GBS during high-temperature tensile deformation. The analysis above agrees well with the conclusion drawn from Figure 6 that the dominant superplastic deformation mechanism of ATZ821 and AT82 alloys is GBS controlled by GB self-diffusion.

## 4. Conclusions

In the present work, the AT82 and ATZ811 alloy sheets with fine grains and uniformly dispersed precipitates were fabricated via a controlled 13-pass rolling process. Room-temperature tensile properties and high-temperature superplasticity were investigated on the two alloys. The main conclusions are drawn as follows:

(1) At room temperature, the substitution of 1 Zn for 1 Sn (wt %) in AT82 alloy results in an increase in elongation from ~14 to ~18% because of the finer and more uniformly dispersed precipitates, accompanied by a decrease in UTS and YS, from ~366 MPa to ~355 MPa and ~270 MPa to ~250 MPa, respectively. According to the contribution from different strengthening mechanisms estimated by simplified models, the higher YS of AT82 is due to the cooperative contribution of finer grains and more Mg_2_Sn particles.
(2) Excellent superplasticity was found in the ATZ811 alloy, which exhibits a tensile elongation of ~510% under a strain rate of 10^−3^ s^−1^ at 573 K, in contrast to the inferior elongation of ~380% for the AT82 alloy. This is because that the incompatible deformation between Mg_2_Sn particles and matrix can cause the formation of cavities during high-temperature tensile deformation. Compared with AT82 alloy, the substitution of 1 Zn for 1 Sn (wt %) results in a decreasing amount and a better uniformity of Mg_2_Sn particles in ATZ811 alloy, thus inhibiting the formation of cavities and leading to an excellent superplasticity.
(3) During tensile deformation, there was no obvious grain growth in both alloys because of the high-volume fraction of second phases located at grain boundaries. Meanwhile, the texture weakening was observed, suggesting that grain boundary sliding (GBS) is the dominant superplastic deformation mechanism, which agrees well with the strain rate sensitivity (*m*) and the activation energy (*Q*) calculations.

## Figures and Tables

**Table 1:** Tensile properties at room (298 K) and elevated (573 K) temperature of rolled and annealed AT82 and ATZ811 alloys in this work.

Alloy	298 K			573 K
	YS/MPa	UTS/MPa	Elongation/%	Fracture strain/%
AT82	$270_{-4}^{+3}$	$366_{-5}^{+4}$	$14_{-1}^{+2}$	$380_{-30}^{+20}$
ATZ811	$250_{-2}^{+2}$	$355_{-2}^{+3}$	$18_{-1}^{+1}$	$510_{-20}^{+10}$

## References

[1] Panicker R., Chokshi A.H., Mishra R.K., Verma R., Krajewski P.E. (2009). Microstructural evolution and grain boundary sliding in a superplastic magnesium AZ31 alloy. Acta. Mater..

[2] Wang H.-Y., Rong J., Yu Z.-Y., Zha M., Wang C., Yang Z.-Z., Bu R.-Y., Jiang Q.-C. (2017). Tensile properties, texture evolutions and deformation anisotropy of as-extruded Mg-6Zn-1Zr magnesium alloy at room and elevated temperatures. Mater. Sci. Eng. A.

[3] Boehlert C.J., Chen Z., Gutiérrez-Urrutia I., Llorca J., Pérez-Prado M.T. (2012). In situ analysis of the tensile and tensile-creep deformation mechanisms in rolled AZ31. Acta. Mater..

[4] Wang Z., Wang J.-G., Chen Z.-Y., Zha M., Wang C., Liu S., Yan R.-F. (2018). Effect of Ce Addition on Modifying the Microstructure and Achieving a High Elongation with a Relatively High Strength of As-Extruded AZ80 Magnesium Alloy. Materials.

[5] Figueiredo R.B., Langdon T.G. (2009). Principles of grain refinement and superplastic flow in magnesium alloys processed by ECAP. Mater. Sci. Eng. A.

[6] Zhang Y., Song L., Chen X., Lu Y., Li X. (2018). Effect of Zn and Ca Addition on Microstructure and Strength at Room Temperature of As-Cast and As-Extruded Mg-Sn Alloys. Materials.

[7] Wu F., Qin C., Zheng Y., Pan W., Ma H., Li T., Ye C., Ma X., Chu Z., Cheng L. (2019). Microstructures, Tensile Properties and Creep Characteristics of as-Extruded AZ91 Magnesium Alloy Containing Si, Ca and Rare Earth Elements. Metals.

[8] Wu K.-C., Chang S.-Y., Yeh J.-W. (2015). Optimizing superplasticity of AZ91−xSn magnesium alloys with competitive grain growth and boundary sliding. Mater. Sci. Eng. A.

[9] Zhang D.-T., Xiong F., Zhang W.-W., Qiu C., Zhang W. (2011). Superplasticity of AZ31 magnesium alloy prepared by friction stir processing. Trans. Nonferr. Metal. Soc..

[10] Kim Y.S., Kim W.J. (2016). Microstructure and superplasticity of the as-cast Mg–9Al–1Zn magnesium alloy after high-ratio differential speed rolling. Mater. Sci. Eng. A.

[11] Zha M., Zhang H.-M., Wang C., Wang H.-Y., Zhang E.-B., Jiang Q.-C. (2017). Prominent role of a high volume fraction of Mg17Al12 particles on tensile behaviors of rolled Mg–Al–Zn alloys. J. Alloys Compd..

[12] Wang H.Y., Zhang E.B., Nan X.L., Zhang L., Guan Z.P., Jiang Q.C. (2016). A comparison of microstructure and mechanical properties of Mg–9Al–1Zn sheets rolled from as-cast, cast-rolling and as-extruded alloys. Mater. Des..

[13] Zhao D., Wang Z., Zuo M., Geng H. (2014). Effects of heat treatment on microstructure and mechanical properties of extruded AZ80 magnesium alloy. Mater. Des..

[14] You S., Huang Y., Kainer K.U., Hort N. (2017). Recent research and developments on wrought magnesium alloys. J. Magnes. Alloy..

[15] Zhou Z., Gu Y., Xu G., Guo Y., Cui Y. (2020). Diffusion research in HCP Mg–Al–Sn ternary alloys. Calphad.

[16] Kim B., Do J., Lee S., Park I. (2010). In situ fracture observation and fracture toughness analysis of squeeze cast AZ51–xSn magnesium alloys. Mater. Sci. Eng. A.

[17] Guo Y., Xuanyuan Y., Lia C., Yang S. (2019). Characterization of Hot Deformation Behavior and Processing Maps of Mg-3Sn-2Al-1Zn-5Li Magnesium Alloy. Metals.

[18] Wang H.Y., Rong J., Liu G.J., Zha M., Wang C., Luo D., Jiang Q.C. (2017). Effects of Zn on the microstructure and tensile properties of as-extruded Mg-8Al-2Sn alloy. Mater. Sci. Eng. A.

[19] Wang Y., Wang Q., Ma C., Ding W., Zhu Y. (2013). Effects of Zn and RE additions on the solidification behavior of Mg–9Al magnesium alloy. Mater. Sci. Eng. A.

[20] Wu L., Cui C., Wu R., Li J., Zhan H., Zhang M. (2011). Effects of Ce-rich RE additions and heat treatment on the microstructure and tensile properties of Mg–Li–Al–Zn-based alloy. Mater. Sci. Eng. A.

[21] Yu Z.P., Zha M., Li Z.H., Wang C., Wang H.Y., Jiang Q.C. (2017). Achieving fine grain structure and superplasticity in AZ91-0.4Sn magnesium alloy using short flow rolling process. Mater. Sci. Eng. A.

[22] Nakata T., Xu C., Suzawa K., Yoshida K., Kawabe N., Kamado S. (2018). Enhancing mechanical properties of rolled Mg-Al-Ca-Mn alloy sheet by Zn addition. Mater. Sci. Eng. A.

[23] Ohno M., Mirkovic D., Schmid-Fetzer R. (2006). Phase equilibria and solidification of Mg-rich Mg–Al–Zn alloys. Mater. Sci. Eng. A.

[24] Tang W.N., Park S.S., You B.S. (2011). Effect of the Zn content on the microstructure and mechanical properties of indirect-extruded Mg–5Sn–xZn alloys. Mater. Des..

[25] Zhang H.-M., Cheng X.-M., Zha M., Li Y.-K., Wang C., Yang Z.-Z., Wang J.-G., Wang H.-Y. (2019). A superplastic bimodal grain-structured Mg–9Al–1Zn alloy processed by short-process hard-plate rolling. Materialia.

[26] Guo F., Zhang D., Wu H., Jiang L., Pan F. (2017). The role of Al content on deformation behavior and related texture evolution during hot rolling of Mg-Al-Zn alloys. J. Alloys Compd..

[27] Koike J., Ohyama R. (2005). Geometrical criterion for the activation of prismatic slip in AZ61 Mg alloy sheets deformed at room temperature. Acta. Mater..

[28] Yuan W., Panigrahi S.K., Su J.Q., Mishra R.S. (2011). Influence of grain size and texture on Hall–Petch relationship for a magnesium alloy. Scr. Mater..

[29] Nie J.F. (2003). Effects of precipitate shape and orientation on dispersion strengthening in magnesium alloys. Scr. Mater..

[30] Kim W.J., Jeong H.G., Jeong H.T. (2009). Achieving high strength and high ductility in magnesium alloys using severe plastic deformation combined with low-temperature aging. Scr. Mater..

[31] Ferdous W., Manalo A., Wong H.S., Abousnina R., AlAjarmeh O.S., Zhuge Y., Schubel P. (2020). Optimal design for epoxy polymer concrete based on mechanical properties and durability aspects. Constr. Build. Mater..

[32] Kim W.J., Park I.B. (2013). Enhanced superplasticity and diffusional creep in ultrafine-grained Mg–6Al–1Zn alloy with high thermal stability. Scr. Mater..

[33] Park S.S., You B.S. (2011). Low-temperature superplasticity of extruded Mg–Sn–Al–Zn alloy. Scr. Mater..

[34] Watanabe H., Mukai T., Kohzu M., Tanabe S., Higashi K. (1999). Effect of temperature and grain size on the dominant diffusion process for superplastic flow in an AZ61 magnesium alloy. Acta. Mater..

[35] Kim W.J., Chung S.W., Chung C.S., Kum D. (2001). Superplasticity in thin magnesium alloy sheets and deformation mechanism maps for magnesium alloys at elevated temperatures. Acta. Mater..

[36] Watanabe H., Fukusumi M., Somekawa H., Mukai T. (2010). Texture and mechanical properties of superplastically deformed magnesium alloy rod. Mater. Sci. Eng. A.

